# Performance and Mechanism of Polycarboxylate Superplasticizer in Red Mud Blended Cementitious Materials

**DOI:** 10.3390/polym17131738

**Published:** 2025-06-22

**Authors:** Lei Yang, Pengfei Wang, Shuqiong Luo, Yaxin Wang, Shengye Xu

**Affiliations:** 1 Henan Key Laboratory of Materials on Deep-Earth Engineering, School of Materials Science and Engineering, Henan Polytechnic University, Jiaozuo 454003, China; yanglei@hpu.edu.cn (L.Y.); thee@home.hpu.edu.cn (P.W.); luoshuqiong@hpu.edu.cn (S.L.); 13629591320@163.com (Y.W.); 2 Guangdong Grid Energy Development Corp., Ltd., Guangzhou 510000, China

**Keywords:** red mud-based cementitious material, polycarboxylate superplasticizer, topology, workability, mechanism

## Abstract

The utilization of red mud by blending it into cement paste is still facing poor workability issues due to the finer particle size and higher water absorption of red mud, which can be solved by the addition of polycarboxylate superplasticizer (PCE) to effectively maintain the working performance. However, the specific mechanisms by which different topologies of PCEs, in terms of water-reducing (WR)- and slump-retaining (SR)-type PCEs, influence red mud blended cement paste require further clarification. This research investigates the effect of WR-PCE and SR-PCE on the rheological properties, mechanical properties, and microscopic morphology of red mud blended cement paste under different red mud contents. The results demonstrated that at saturated dosages of 0.5% WR-PCE and 0.75% SR-PCE, both types of PCEs improved paste fluidity and reduced plastic viscosity and shear stress. Moreover, the time-dependent fluidity loss rate of the SR-PCE-incorporated paste was lower to that of the WR-PCE-incorporated paste at 30 and 60 min. With 0% and 25% red mud contents, the compressive strengths at 1, 3, 7, and 28 days were higher for WR-PCE than for SR-PCE due to the enhanced hydration of C_2_S and C_3_S. Furthermore, hydration products in the WR-PCE-incorporated paste were more uniformly distributed compared to the SR-PCE-incorporated paste. However, a 50% red mud content negatively impacted paste strength, likely due to the high alkalinity destabilizing the PCE. This study aims to elucidate the mechanistic relationship between PCE topology and the improved performance of red mud blended cement paste.

## 1. Introduction

Red mud, a strongly alkaline solid waste residue generated in the industrial smelting of alumina, has an annual emission of 146 million tons but a utilization rate of only 7% [1]. Currently, red mud is primarily treated through landfill and stockpiling, practices that consume vast land resources and lead to substantial environmental pollution. Its primary components, including SiO_2_, CaO, Al_2_O_3_, and Fe_2_O_3_, can be used to produce cementitious materials, thus enabling large-scale utilization [2]. However, due to the fine particle size of red mud (1–20 μm), it has a high water absorption rate, which significantly reduces the fluidity performance and mechanical properties of cement concrete when incorporated [1,3]. This limitation restricts its widespread application in cementitious materials [4]. Incorporating high-efficiency superplasticizers is currently a proven approach to ensure the working performance of cementitious materials derived from red mud [4].

Compared with traditional high-efficiency superplasticizers (naphthalene sulfonate, melamine sulfonate), polycarboxylate superplasticizer (PCE) offers advantages such as low dosage, high water reduction, and environmental friendliness [5,6,7], making it indispensable in modern concrete preparation [8]. PCE is generally prepared through free radical polymerization of small acrylic monomers and large polyoxyethylene ether (or ester) monomers in aqueous solution, forming a typical comb-like molecular structure with a polyethylene oxide (PEO) side chain and a negatively charged main chain [9]. When added to cement paste, PCE quickly attaches to cement particles under robust electrostatic interactions between the -COO- groups in the copolymer skeleton and the highly charged mineral surfaces. This includes complexation between the -COO- groups and Ca^2+^ ions in the pore solution [10]. Meanwhile, the PEO side chains extend into the pore solution, creating a molecular adsorption layer [11]. Furthermore, the molecular structure of PCE directly influences the yield stress, strength progression, and microstructural changes in freshly mixed concrete [12,13].

At present, innovative strategies centered on the development and modification of PCE molecular structures have been adopted to improve the rheological properties, adhesion properties, and durability of cement paste and concrete [14,15]. Some investigations have concentrated on altering the structure of PCE. For instance, Fan et al. [16] replaced some of the carboxyl groups with trialkoxysilanes to increase PCE’s resistance to sulfate ions. Nonetheless, in engineering applications, long-distance transport of concrete and high ambient temperatures are common, leading to unsatisfactory slump preservation of traditional PCE [17]. Consequently, PCE still requires certain architectural innovations to achieve significant performance improvements, and research on slump-retaining PCE (SR-PCE) has been initiated. Zhou et al. [18] discovered that SR-PCE with an altered topology design exhibits a better operational performance compared to conventional water-reducing PCE (WR-PCE). Nonetheless, researchers have primarily applied SR-PCE to cement clear mortar or concrete [19,20], with limited research on its mechanism of action in red mud blended cement paste. Does SR-PCE still exhibit superior workability over conventional WR-PCE, and is there a difference in adsorption between SR-PCE and conventional WR-PCE on the surface of red mud blended cement paste? How do the rheological and mechanical properties change? To answer these questions, this study investigates the impact of WR-PCE and SR-PCE on the rheological properties, mechanical properties, and microscopic morphology of red mud blended cement paste with various red mud contents. The aim is to establish the mechanism of action of PCE with different molecular structures in red mud blended cement paste, thereby offering a theoretical foundation for effectively utilizing red mud.

## 2. Materials and Methods

### 2.1. Raw Materials

The cement utilized was P.O 42.5 ordinary silicate cement from Jiaozuo Qianye Cement Co., Ltd, China. The red mud was Bayer red mud from Zhongzhou Aluminium Co., Ltd., in Henan, Jiaozuo, China. Table 1 presents the chemical compositions of the red mud. Initially, it was naturally dried, then crushed using a crusher, and subsequently dried to a stable weight in a vacuum-drying oven at 45 ± 2 °C for 48 h. After drying, the red mud was passed through a 75 μm sieve to produce the desired red mud powder. Deionized water was used for all experiments.

**Figure 1:**
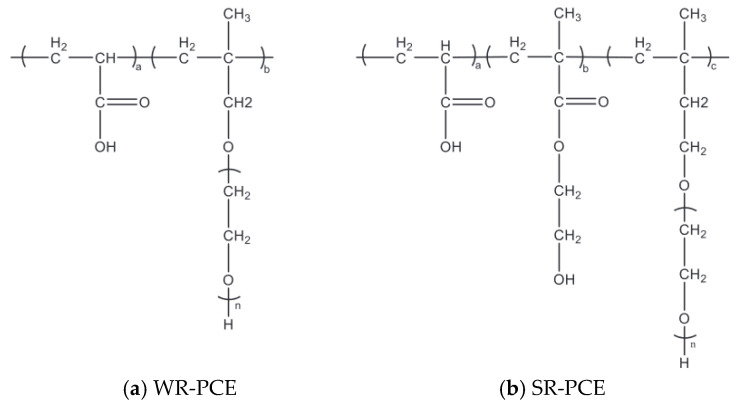
Chemical structures of PCEs: (**a**) WR-PCE, where “n” represents the average degree of polymerization of the PEO side chains; (**b**) SR-PCE, where “a”, “b”, and “c” represent the molar ratios of the respective monomer units.

The materials used for the preparation of PCE include methyl allyl polyoxyethylene ether (>99% purity), isoprenyl oxypoly (ethylene glycol) (>99% purity), 2-hydroxyethyl methacrylate (>99% purity), acrylic acid (AA, >99% purity), sodium hydroxide (>98% purity), vitamin C (Vc, >99% purity), mercaptopropionic acid (MPA, >99% purity), and hydrogen peroxide solution (>99% purity). Figure 1 depicts the chemical structures of WR-PCE and SR-PCE. The WR-PCE values, corresponding to the areas belonging to Ha and Hb, were 3.00 and 6.36, respectively. Consequently, the copolymerization ratio of AA to HPEG was approximately 4.37:1.00. Regarding the SR-PCE areas, those of Ha, Hb, and Hc were 1.14, 4.00, and 2.82, respectively. The copolymerization ratios of AA, HEMA, and TPEG were calculated to be 7.42:5.26:1.00 [21].

### 2.2. Synthesis of PCEs

In this study, we synthesized WR-PCE and SR-PCE, and the synthesis process is described as follows: An example is the synthesis of WR-PCE. The experiment consisted of securing a four-necked flask to a stand and configuring a stirring blade and thermometer at the mouth of the flask. Methyl allyl polyoxyethylene ether was added to a kettle at the bottom of the flask along with an appropriate amount of deionized water, stirring until the macromonomers dissolved. Then, 30% H_2_O_2_ (mol/L) was added and stirred for 10 min, and the peristaltic pump was opened. At the same time, liquids A and B were pumped into the flask. Liquid A consisted of an aqueous solution of AA and deionized water; liquid B consisted of an aqueous solution of MPA, Vc, and deionized water. Liquid A was added over 40 min, liquid B was added over 50 min, and the temperature was maintained for 30 min after adding liquid A. At the end of the heat preservation, 30% liquid alkali (NaOH, 0.750 mol/L) was introduced to adjust the pH level to between 6 and 7, followed by the addition of deionized water to achieve polycarboxylate superplasticizer liquid with a solid content of 40%. For SR-PCE, the macromonomer was replaced with isoprenyl oxypoly (ethylene glycol), and 2-hydroxyethyl methacrylate mixed with acrylic acid was added and stirred, following the same steps to obtain SR-PCE.

### 2.3. Preparation of Cement Specimens

The red mud blended cement cementitious material (400 g, the water-to-binder ratio was 0.4) and water-reducer mixture (160 g of water and water-reducer mixed uniformly) were weighed. The material was mixed with a collodion mixer at slow speed for 2 min, allowed to rest for 15 s, then mixed at fast speed for 2 min. After thorough and homogeneous mixing, the paste was carefully poured into cubic molds 2 cm in size. For each mixture, 12 specimens were fabricated to conduct compressive strength tests at 1, 3, 7, and 28 days [22], after vibration. The molds were covered with cling film, marked well, and kept in the standard curing room to cure for 24 h. After 24 h, the specimens were removed from the molds and returned to the curing room to continue curing until they reached the required age for testing.

In total, 300 g of red mud blended cement cementitious material was weighed. The cementitious material was mixed with superplasticizers (dosages of 0%, 0.1%, 0.2%, 0.3%, 0.5%, 0.75%, and 1%), using a water/cement ratio of 0.4. The cementitious material was mixed with the superplasticizers using a cement sand mixer at a slow speed for 2 min, allowed to rest for 15 s, then mixed at a fast speed for another 2 min. After mixing well, the cementitious material was poured into truncated cone molds and tested for fluidity to determine the WR-PCE and SR-PCE saturated dosing.

### 2.4. Test

The experiment referred to the stipulations of GB/T 1346-2011 [23] to test the paste’s initial and final setting times. The fluidity of the specimens was determined using China Shanghai Anton Paar’s MCR 302 SN82880383 dynamic shear rheometer, following the stipulations of GB/T 8077-2023 [24]. The rotor ST24-2D/2V/2V-30 with serial number SN51403 was chosen. The shear rate was configured to range from 0 to 120 revolutions per minute (r/min) during the ascending phase and from 120 to 0 r/min during the descending phase. A total of 60 data points were acquired, and the measurement duration was 100 s. The specimens’ compressive strength was quantified using the YNS-Y1000 microcomputer-controlled electronic pressure tester from China Changchun Institute of Mechanical Engineering with a constant loading rate of 0.2 kN/s.

The samples’ physical composition was analyzed using a Smart-Lab X-ray diffractometer from Rigaku, Tokyo, Japan. XRD patterns were obtained in the 5–70° range at a scanning rate of 2°·min^−1^ using a Cu target. The microstructure was examined with a Merlin Compact Field Emission Scanning Electron Microscope (FESEM) from Zeiss, Oberkochen, Germany. The samples’ thermal properties were assessed utilizing the programmed enthalpy analysis and mass spectrometry (EA-MS) system (Model STA449F3-QMS403D) from NETZSCH, Waldkraiburg, Germany.

## 3. Results

### 3.1. Determination of Saturated Superplasticizer Dosing Points

**Figure 2:**
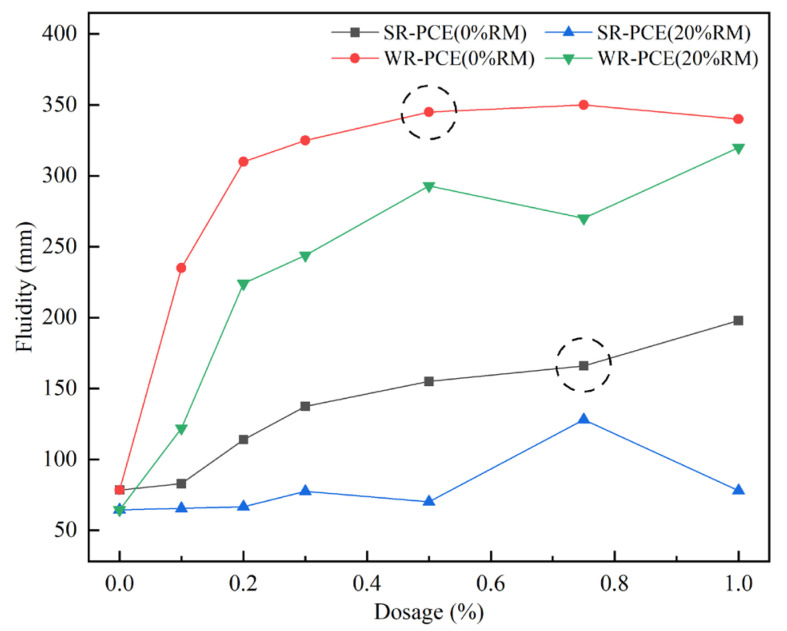
Effect of superplasticizer dosage on paste fluidity with various red mud dosages.

Figure 2 illustrates the variations in the fluidity of red mud blended cement paste incorporating SR-PCE and WR-PCE at red mud concentrations of 0% and 20%. It was observed that at 0% red mud content, the paste fluidity increased with higher dosages of the superplasticizer. The fluidity changes became negligible when the dosages exceeded 0.75% for SR-PCE and 0.5% for WR-PCE. Consequently, the saturation dosages were determined to be 0.75% for SR-PCE and 0.5% for WR-PCE. At a red mud content of 20%, the fluidity of the paste containing 0.75% SR-PCE improved by 98.4% compared to the paste without PCE, while the fluidity of the paste with 0.5% WR-PCE increased by 354.3%. This enhancement is attributed to PCE molecules dissociating into macromolecular anions in water, which then adhere to the surfaces of cement particles and their hydration products. This adsorption increases the electrostatic repulsion between particles, disrupts and prevents the formation of flocculated structures in the paste, and increases the amount of free water, resulting in improved paste fluidity [25]. When the dosages of the superplasticizer and red mud were kept constant, the paste with WR-PCE consistently exhibited greater fluidity than that with SR-PCE. Thus, at a fixed red mud dosage of 20%, WR-PCE proved to be more effective than SR-PCE in enhancing the fluidity of red mud blended cement paste.

### 3.2. Rheological Property Analysis

#### 3.2.1. Setting Time of Red Mud Blended Cement Paste

**Figure 3:**
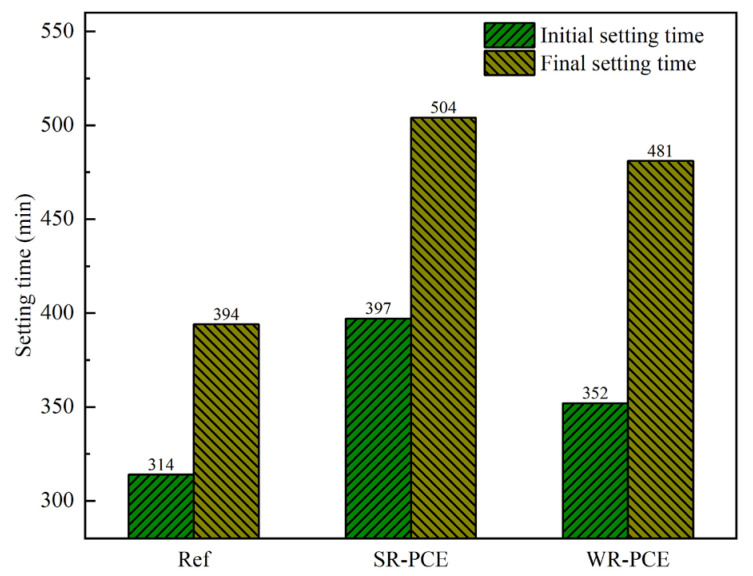
Setting time of red mud blended cement paste with a 25% red mud dosage.

When the red mud admixture was maintained at 25%, the setting time of the reference group, SR-PCE (0.75%), and WR-PCE (0.5%) red mud blended cement paste was measured, as presented in Figure 3. The initial setting time for SR-PCE and WR-PCE was 26.4% and 12.1% longer than the reference group, respectively, while the final setting time was 27.9% and 22.1% longer. These results indicate that PCE retards the setting time of red mud blended cement paste, with SR-PCE exhibiting a stronger retarding effect compared to WR-PCE. This is attributed to SR-PCE having longer and denser side chains than WR-PCE, which enhances spatial site resistance and thus more effectively delays cement hydration [26].

#### 3.2.2. Influence of Superplasticizers on the Fluidity Loss of Red Mud Blended Cement Paste

**Figure 4:**
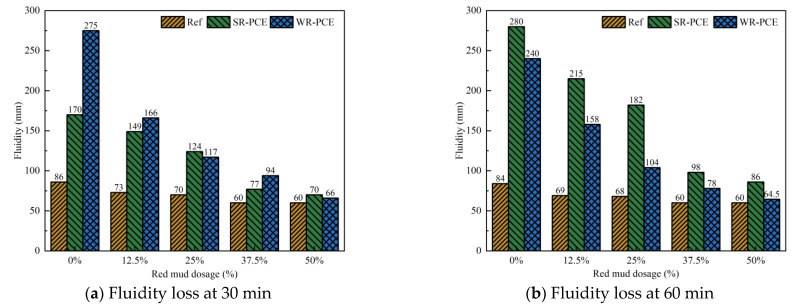
Impact of different superplasticizers on the fluidity loss of paste (reference group, 0.75% SR-PCE, and 0.5% WR-PCE) with various red mud dosages.

Figure 4 illustrates how SR-PCE and WR-PCE affect the fluidity loss of red mud blended cement paste with varying red mud dosages (0%, 12.5%, 37.5%, and 50%), while Table 2 details the impact of superplasticizers on the decrease rate of fluidity loss relative to a 0% red mud dosage. As the red mud dosage increased, the fluidity of the reference, SR-PCE, and WR-PCE groups exhibited a declining trend at both 30 and 60 min. The fluidity of the reference group fell below 100 mm, indicating excessive viscosity. For equal amounts of red mud, the SR-PCE and WR-PCE groups showed higher flow than the reference group, indicating that PCE enhances the initial flow of freshly mixed red mud blended cement paste. This improvement is attributed to the lubricating effect of PCE, which forms hydrogen bonds with water due to its hydrophilic carboxyl and other polar groups, and weakens the van der Waals forces between red mud, cement, and PCE, thereby creating steric repulsion that disperses red mud and cement particles [27].

At a red mud dosage of 12.5%, the SR-PCE red mud blended cement paste experienced the smallest fluidity loss compared to WR-PCE for both 30 and 60 min, showing reductions of 12.4% and 23.2%, respectively (Table 2). This is due to the ester groups in SR-PCE hydrolyzing in the alkaline environment of cement hydration, releasing carboxyl groups which significantly reduce the fluidity loss [19]. When the red mud dosage exceeded 12.5%, the fluidity loss for both SR-PCE and WR-PCE red mud blended cement paste increased by more than 25% at both 30 and 60 min. This occurred because the extensive specific surface area of red mud decreases the amount of free water in the paste. Additionally, red mud adsorbs PCE, diminishing its efficacy on the cement particle surfaces [28].

#### 3.2.3. Influence of PCE on the Shear Stress of Red Mud Blended Cement Paste

**Figure 5:**
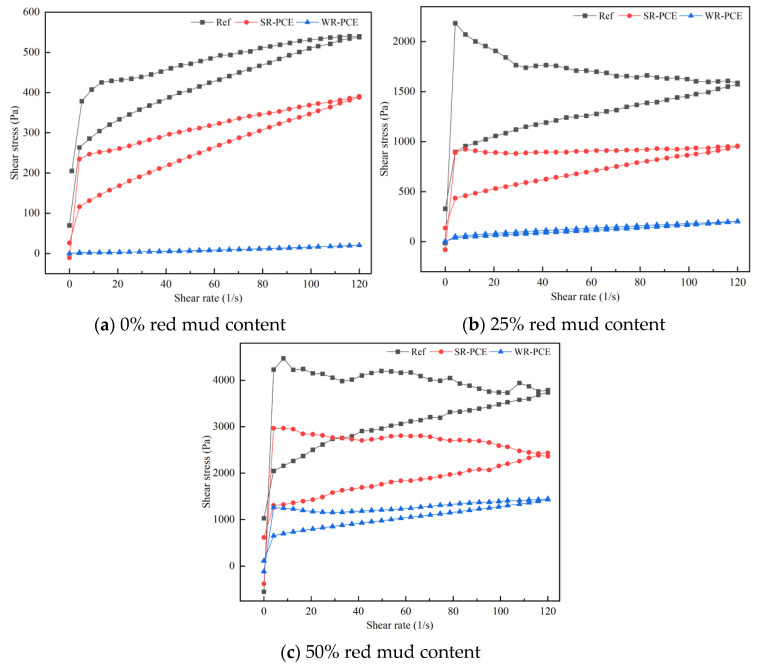
Hysteresis curves of paste with various red mud dosages (0%, 25%, and 50%) under PCE action.

Figure 5 presents the hysteresis curves for red mud blended cement paste with red mud contents of 0%, 25%, and 50% under the influence of superplasticizers. Table 3 details the yield stress values for these paste. When no red mud was incorporated (0%), the shear stress of the red mud blended cement paste increased with the shear rate in both the reference group and the groups containing SR-PCE and WR-PCE. However, the increase in shear stress was notably smaller for the WR-PCE group. The Bingham model was used to characterize the rheological behavior [29]. At 0% red mud, both the reference group and the SR-PCE-incorporated paste behaved as standard Bingham fluids, whereas the WR-PCE-incorporated paste exhibited properties closer to a Newtonian fluid. At a red mud content of 25%, the shear stress of the SR-PCE and WR-PCE-incorporated paste also increased with the shear rate. The WR-PCE-incorporated paste behaved similarly to a Newtonian fluid, while the reference group and SR-PCE-incorporated paste continued to act as Bingham fluids. The WR-PCE-incorporated paste showed the lowest yield stress, approaching zero. When the content reached 50%, the overall shear stress rose linearly with the shear rate. Both the reference group and the PCE-incorporated paste remained Bingham fluids, with the WR-PCE-incorporated paste consistently exhibiting the lowest yield stress.

#### 3.2.4. Influence of Superplasticizers on the Apparent Viscosity of Red Mud Blended Cement Paste

**Figure 6:**
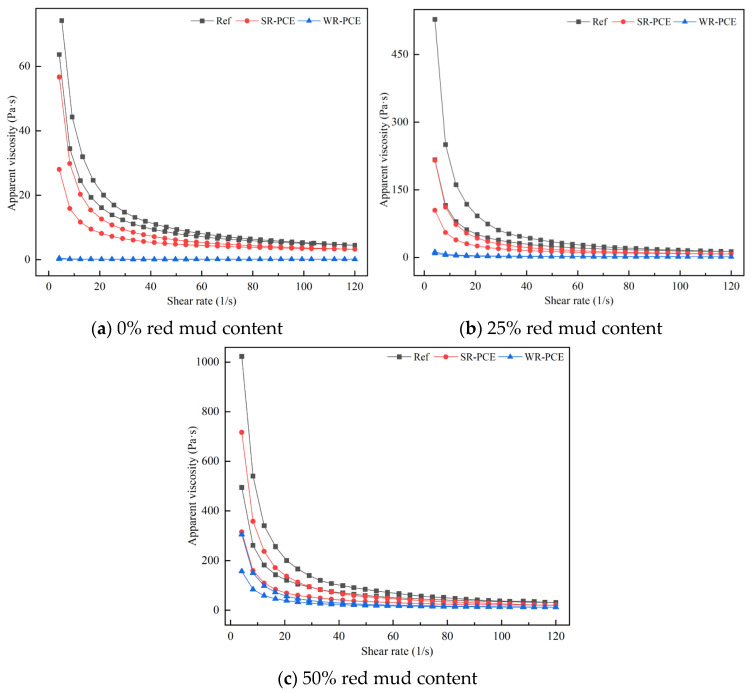
Impact of PCE on the apparent viscosity of red mud blended cement paste with red mud contents of 0%, 25%, and 50%.

Figure 6 illustrates how PCE affects the apparent viscosity of red mud blended cement paste with red mud contents of 0%, 25%, and 50%, while Table 4 presents the impact of PCE on these pastes’ plastic viscosity. When the red mud content was 0% or 25%, the apparent viscosity of the reference group and the SR-PCE-incorporated paste increased with the shear rate. In contrast, the WR-PCE-incorporated paste showed minimal fluctuations in apparent viscosity. Once stabilized, the viscosities of all three pastes approached zero, representing the plastic viscosity, with the order of magnitude being as follows: reference group > SR-PCE > WR-PCE. When the red mud content was 50%, the apparent viscosity of the reference group, SR-PCE-incorporated paste, and WR-PCE-incorporated paste decreased with increasing shear rate. Upon stabilization, the viscosities of all three pastes again approached zero, indicating plastic viscosity, with the same order of magnitude: reference group > SR-PCE > WR-PCE. This observation aligns with the findings in Section 3.2.3.

### 3.3. Compressive Strength Analysis

**Figure 7:**
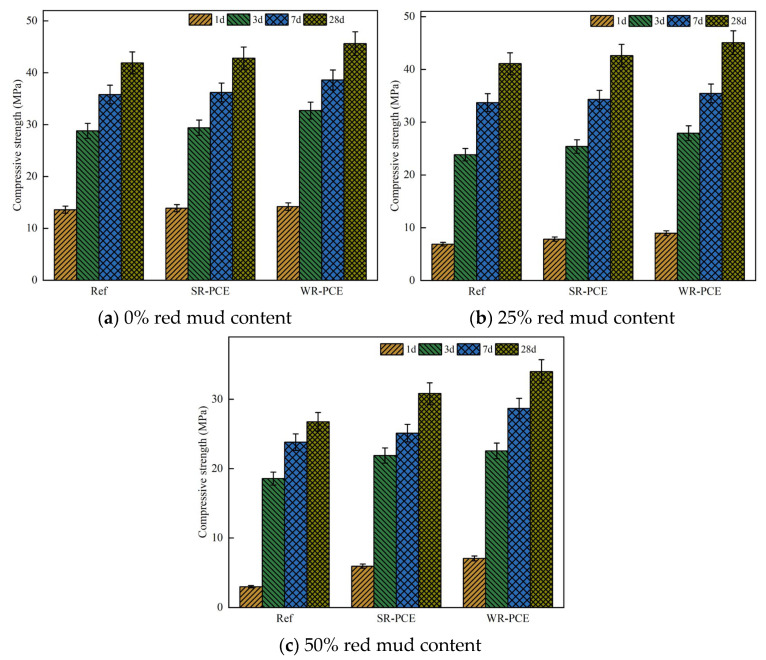
Impact of PCE on the compressive strength of paste with various red mud contents.

Figure 7 displays the specimens’ compressive strength at 1, 3, 7, and 28 days, with superplasticizers added at 0%, 25%, and 50% red mud contents. The data reveal that the compressive strength of the PCE-enhanced paste was marginally greater than that of the reference group across all red mud contents (0%, 25%, and 50%). This suggests that the addition of PCE not only does not compromise the compressive strength of red mud blended cement paste but also has a slight strengthening effect. The enhancement can be attributed to the disruption of the flocculation state among cement particles by PCE. The anionic PCE molecules attach to the surfaces of red mud and cement particles, creating electrostatic repulsion. The spatial resistance effect of PCE’s long side chains further disperses the cement particles, freeing up water, which then participates in the hydration process, resulting in more hydration products [30].

Additionally, the carboxylic acid groups in PCE easily complex with Ca^2+^ in the red mud blended cement paste, which reduces the pore size distribution, fills internal pores, and enhances the overall strength of the specimens [31]. The early and late strengths of the WR-PCE samples were greater than those of the SR-PCE samples. This might be because the dispersion properties of WR-PCE in red mud blended cement paste are superior, further promoting the hydration reaction [11].

When the red mud content was increased to 50%, the overall compressive strength decreased significantly compared to the 0% and 25% red mud content paste. This reduction could be due to the high alkalinity of the paste at higher red mud dosages, which destabilizes the PCEs in such highly alkaline environments [32].

### 3.4. Mineral Composition Analysis

**Figure 8:**
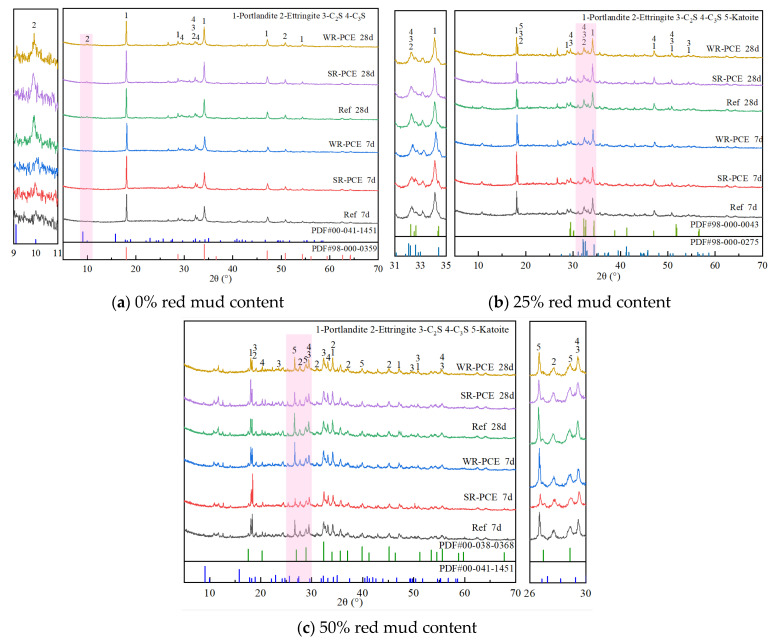
XRD plots of paste with different ages and different red mud dosages.

Figure 8 presents the X-ray diffraction (XRD) plots of red mud blended cement paste with red mud contents of 0%, 25%, and 50% at various ages. At both 7 and 28 days, with a red mud content of 0% (Figure 8a), no significant difference was observed in the types of hydration products between the reference group and the paste incorporated with SR-PCE and WR-PCE. This indicates that PCE doping does not alter the hydration product types in the cement paste but enhances its physical properties by reducing the water/cement ratio while maintaining its strength. Moreover, compared to the 7-day samples, the 28-day samples exhibited higher peak intensities of C-S-H gel and Ca (OH)_2_, along with decreased peaks of C_2_S and C_3_S. This suggests that SR-PCE and WR-PCE accelerate the hydration process, depleting C_2_S and C_3_S and generating more C-S-H hydration products.

For red mud contents of 25% and 50% (Figure 8b,c), the 28-day samples with SR-PCE and WR-PCE showed higher peak intensities of C-S-H and Ca (OH)_2_ compared to the samples without water-reducers. This indicates that SR-PCE and WR-PCE enhance the hydration degree of C_2_S and C_3_S over time, producing more C-S-H and hence improving strength. Additionally, the presence of hydrogrossular (Ca_3_Al_2_ (SiO_4_) _3−x_ (OH)_4x_) peaks is due to the red mud addition. The intensity of hydrogrossular peaks decreased in the 28-day samples compared to the 7-day samples, suggesting that red mud participates in the hydration reaction, which is not complete [33]. In the in-depth analysis of the diffraction patterns, the peak position of Ca (OH)_2_ in the samples doped with WR-PCE exhibited a subtle shift. This phenomenon could be attributed to the dispersion effect imparted by WR-PCE and its impact on the crystallization process of the hydration products. Specifically, it might have led to minor lattice distortions or alterations in the integrity of its crystallization. Furthermore, the broadening of certain peaks was also noted. This can be associated with multiple factors, such as the crystal structure, sample inhomogeneity, and physical transformations [34].

### 3.5. Micro-Morphological Analysis

**Figure 9:**
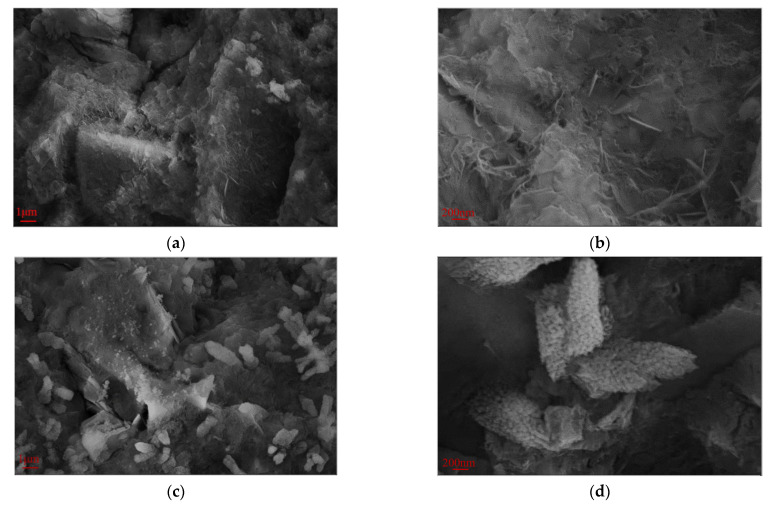

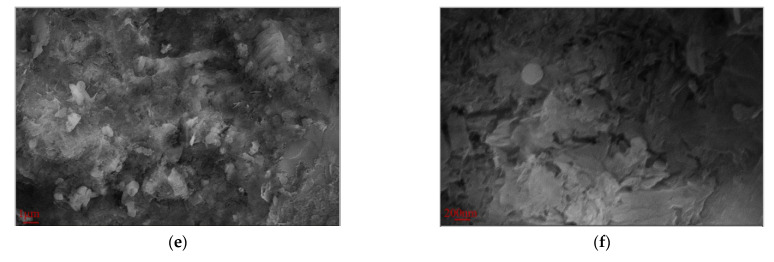
SEM plots at 28 d for the hardened red mud blended cement paste from the reference group, SR-PCE, and WR-PCE with a 25% red mud dosage. (**a**) SEM image at 28 d of hardened paste from the reference group (×5000); (**b**) 28 d SEM image of hardened paste from the reference group (×20,000); (**c**) 28 d SEM image of hardened SR-PCE-incorporated paste (×5000); (**d**) 28 d SEM image of hardened SR-PCE-incorporated paste (×20,000); (**e**) 28 d SEM image of hardened WR-PCE-incorporated paste (×5000); (**f**) 28 d SEM image of hardened WR-PCE-incorporated paste (×20,000).

Figure 9 presents the SEM images at 28 days (magnifications of ×5000 and ×20,000) for the hardened red mud blended cement paste from the reference group, SR-PCE, and WR-PCE with a 25% red mud dosage. Figure 9a,b show a substantial formation of fibrous C-S-H gels and flaky Ca (OH)_2_ crystals. The fibrous C-S-H gels not only adhered to the flaky Ca (OH)_2_ but also effectively filled the pores, densifying the matrix structure. At a magnification of ×5000 (Figure 9c), numerous uniformly distributed flocculated structures were observed. These structures were adsorbed onto the surfaces of cement particles and were evenly distributed throughout the cement paste.

At a magnification of ×20,000 (Figure 9d), notable differences in the morphology of the hydration products in the paste incorporated with SR-PCE were observed when compared to the reference group. The C-S-H gel appeared less well-developed and more aggregated. This morphological alteration is likely an indirect consequence of the adsorption of SR-PCE molecules onto the particle surfaces. Such adsorption influences the nucleation and growth processes of the C-S-H gel surface, effectively encapsulating the gel.

The paste incorporated with WR-PCE exhibited significantly lower yield stress and plastic viscosity, providing quantitative evidence of its superior dispersing effect. The SEM image in Figure 9e reveals the microstructural implications of this enhanced dispersion: the hydration products form a more homogeneous and interconnected network structure throughout the paste. This observable uniformity at the microscale is consistent with and corroborates the superior workability measured at the macroscale.

Finally, the SEM image in Figure 9f at a magnification of ×20,000 shows that the C-S-H gel in the paste incorporated with WR-PCE exhibits a network structure, which differs from the fibrous structure observed in the reference group.

### 3.6. Thermal Analysis

**Figure 10:**
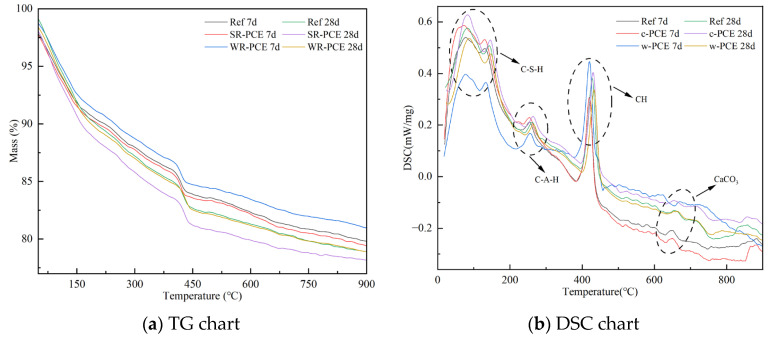
TG and DSC plots of hardened paste in the reference, SR-PCE, and WR-PCE groups (7 and 28 days).

Figure 10 presents the thermogravimetric analysis (TG) and differential scanning calorimetry (DSC) plots for the reference group, SR-PCE, and WR-PCE red mud blended cement paste with a 25% red mud admixture. Table 5 details the mass loss of hydration products for 7- and 28-day samples from the reference group, SR-PCE, and WR-PCE red mud blended cement paste across three stages: C-S-H gel dehydration [35], Ca (OH)_2_ crystal dehydration [36], and CaCO_3_ decomposition [37]. The TG and DSC plots (Figure 10a,b) showed a mass loss up to 100 °C, primarily due to the free and adsorbed water’s natural evaporation in the paste [38]. From 100 to 300 °C, mass loss is mainly attributed to C-S-H gel dehydration, with WR-PCE-incorporated samples exhibiting greater C-S-H dehydration, indicating more C-S-H formation. Between 400 and 500 °C, the mass loss corresponds to the Ca(OH)_2_ crystal dehydration, and from 600 to 750 °C, it is caused by CaCO_3_ decomposition. Additionally, the mass loss of Ca(OH)_2_ crystals in the 28-day hydration products of the reference group, SR-PCE, and WR-PCE red mud blended cement paste was lower than in the 7-day samples. This indicates that more Ca(OH)_2_ crystals are consumed as samples age, leading to increased generation of C-S-H gels.

### 3.7. Mechanistic Analysis

The results from the rheological property experiments, compressive strength experiments, and XRD, SEM, and TG-DSC analyses reveal the underlying mechanisms in the early stages of the red mud blended cement paste reaction with WR-PCE and SR-PCE. The hydration of tricalcium aluminate (C_3_A) and tetracalcium ferroaluminate (C_4_AF) in the cement imparts a positive charge on the surface of cement particles. This enhances the adsorption of hydrolyzed PCE molecules, which generate carboxylate and other anions. The hydration product Ca (OH)_2_ and the alkaline nature of red mud favor the PCE adsorption on the red mud and cement particle surfaces [39].

As numerous PCE molecules adsorb onto the surfaces of red mud and cement particles, these surfaces become negatively charged. This leads to electrostatic repulsion between red mud and cement particles [10], dispersing them and extending the red mud blended cement paste’s initial and final setting times [40]. The hydration products in the WR-PCE-incorporated red mud blended cement paste were more evenly distributed compared to those with SR-PCE. This is primarily due to the complexation of SR-PCE with Ca^2+^ in the paste and the cross-linking between SR-PCE molecules, which diminishes its dispersibility [41]. However, the pivotal characteristic of SR-PCE lies in its sustained-release mechanism achieved through ester hydrolysis. As time progresses, within the high-pH environment of cement paste, the ester groups in SR-PCE undergo gradual hydrolysis, thereby liberating new carboxylate groups. These newly generated functional groups continuously adsorb onto cement particles and their hydration products. This process replenishes the dispersing force and counteracts the slump loss resulting from the ongoing hydration process. Consequently, although the initial fluidity enhancement of SR-PCE is modest, its long-term slump retention is remarkable. This two-stage mechanism effectively reconciles its initial dispersibility with its outstanding slump-retaining performance. Consequently, the apparent viscosity and shear stress of the WR-PCE red mud blended cement paste are lower, resulting in greater fluidity.

Moreover, SR-PCE molecules possess longer and denser side chains compared to WR-PCE, which enhances their spatial site resistance effect, thereby more effectively retarding cement hydration [27]. This results in a lower rate of time-dependent loss and better retardation and slump retention effects.

## 4. Conclusions

### The Primary Conclusions Can Be Drawn as Follows:

(1) At the saturated dosages of WR-PCE and SR-PCE of 0.5% and 0.75%, respectively, the addition of PCEs improved the fluidity of the paste. The SR-PCE-incorporated paste exhibited a lower rate of time-dependent fluidity loss at 30 and 60 min compared to the WR-PCE-incorporated paste. With 0% and 25% red mud contents, the 1-day, 3-day, 7-day, and 28-day compressive strengths for pastes incorporated with WR-PCE and SR-PCE were higher than those of the reference group. This is due to the enhanced hydration reaction of both C_2_S and C_3_S facilitated by WR-PCE and SR-PCE, with WR-PCE showing a more pronounced effect.
(2) At 0% and 25% red mud contents, the yield stress of the WR-PCE-incorporated paste approached to near zero, resembling a Newtonian fluid with optimal dispersibility. In contrast, the reference group and the SR-PCE-incorporated paste behaved as Bingham fluids. The plastic viscosity order was as follows: reference group > SR-PCE > WR-PCE. As the red mud content increased, the paste’s shear stress and apparent viscosity increased. When the red mud content reached 50%, all pastes exhibited Bingham fluid characteristics, maintaining the following plastic viscosity order: reference group > SR-PCE > WR-PCE.
(3) In both unincorporated and PCE-incorporated red mud blended cement paste, C-S-H appeared fibrous and network-like, while Ca (OH)_2_ was flaky. However, the WR-PCE-incorporated paste showed a more even hydration product distribution compared to the SR-PCE-incorporated paste. A high red mud content of 50% negatively affected its strength, likely due to the excessive alkalinity destabilizing the PCE.

## Figures and Tables

**Table 1:** Chemical composition of cement and red mud (wt%).

Raw Material	SiO_2_	MgO	Al_2_O_3_	CaO	Fe_2_O_3_	K_2_O	Na_2_O	LOI
Cement	21.75	3.76	5.57	63.62	4.07	0.33	0.07	-
Red mud	20.32	0.70	23.38	11.63	16.35	0.62	7.24	14.25

Note: LOI represents loss on ignition.

**Table 2:** Impact of superplasticizers on the decrease rate of fluidity loss through time of red mud blended cement paste (compared to a 0% red mud dosage).

Red Mud	Time (min)	12.5%	25.0%	37.5%	50.0%
PCE					
SR-PCE	30	12.4%	27.1%	54.7%	58.8%
WR-PCE	30	39.6%	57.5%	65.8%	76.0%
SR-PCE	60	23.2%	35.0%	66.4%	69.2%
WR-PCE	60	34.2%	56.7%	67.5%	73.1%

**Table 3:** Yield stress of red mud blended cement paste with various red mud dosages under the action of different superplasticizers.

	Yield Stress/Pa (Red Mud 0%)	Yield Stress/Pa (Red Mud 25%)	Yield Stress/Pa (Red Mud 50%)
Ref	69.678	327.730	1026.300
SR-PCE	26.193	136.780	612.500
WR-PCE	0.015	1.811	112.660

**Table 4:** Impact of PCE on the plastic viscosity of red mud blended cement paste with red mud contents of 0%, 25%, and 50%.

	Plastic Viscosity/Pa·s (Red Mud 0%)	Plastic Viscosity/Pa·s (Red Mud 25%)	Plastic Viscosity/Pa·s (Red Mud 50%)
Ref	4.499	13.228	31.548
SR-PCE	3.254	7.966	20.288
WR-PCE	0.172	1.697	12.050

**Table 5:** Mass loss of 7 and 28 d hydration products of the reference group, SR-PCE, and WR-PCE hardened paste at each hydration stage.

	Curing Time (d)	Stage I	Stage II	Stage III	Total Loss
Ref	7	2.7%	5.7%	5.2%	18.4%
SR-PCE	7	3.3%	4.8%	5.4%	19.0%
WR-PCE	7	4.7%	4.3%	5.3%	19.5%
Ref	28	3.5%	5.2%	5.5%	19.6%
SR-PCE	28 d	4.4%	4.6%	5.0%	20.2%
WR-PCE	28 d	5.2%	4.0%	4.1%	20.9%

## Data Availability

The original contributions presented in this study are included in the article. Further inquiries can be directed to the corresponding author.
